# Complex nature of apparently balanced chromosomal rearrangements in patients with autism spectrum disorder

**DOI:** 10.1186/s13229-015-0015-2

**Published:** 2015-03-25

**Authors:** Anne-Claude Tabet, Alain Verloes, Marion Pilorge, Elsa Delaby, Richard Delorme, Gudrun Nygren, Françoise Devillard, Marion Gérard, Sandrine Passemard, Delphine Héron, Jean-Pierre Siffroi, Aurelia Jacquette, Andrée Delahaye, Laurence Perrin, Céline Dupont, Azzedine Aboura, Pierre Bitoun, Mary Coleman, Marion Leboyer, Christopher Gillberg, Brigitte Benzacken, Catalina Betancur

**Affiliations:** Department of Genetics, AP-HP, Robert Debré University Hospital, 48 boulevard Sérurier, 75019 Paris, France; INSERM, UMR 1130, Neuroscience Paris Seine, 9 quai Saint Bernard, 75005 Paris, France; CNRS, UMR 8246, Neuroscience Paris Seine, 9 quai Saint Bernard, 75005 Paris, France; Sorbonne Universités, UPMC Univ Paris 6, Institut de Biologie Paris-Seine, 9 quai Saint Bernard, 75005 Paris, France; INSERM, UMR 1141, Robert Debré University Hospital, 48 boulevard Sérurier, 75019 Paris, France; Department of Child and Adolescent Psychiatry, AP-HP, Robert Debré University Hospital, 48 boulevard Sérurier, 75019 Paris, France; Fondation Fondamental, 40 rue de Mesly, 94000 Créteil, France; Gillberg Neuropsychiatry Centre, University of Gothenburg, Kungsgatan 12, 41119 Göteborg, Sweden; Département de Génétique et Procréation, CHU de Grenoble, Hôpital Couple-Enfant, avenue du Maquis du Grésivaudan, 38043 Grenoble, France; Neurology Unit, AP-HP, Robert Debré University Hospital, 48 boulevard Sérurier, 75019 Paris, France; Medical Genetics Unit, AP-HP, Pitié-Salpêtrière University Hospital, 47 boulevard de l’Hôpital, 75013 Paris, France; Service de Génétique et d’Embryologie Médicales, AP-HP, Trousseau Hospital, 26 avenue du Docteur Arnold Netter, 75012 Paris, France; Cytogenetics Unit, AP-HP, Jean Verdier Hospital, allée du 14 Juillet, 93140 Bondy, France; Paris 13 University, Sorbonne Paris Cité, UFR SMBH, 74 rue Marcel Cachin, 93000 Bobigny, France; Medical Genetics Unit, AP-HP, Jean Verdier Hospital, allée du 14 Juillet, 93140 Bondy, France; Foundation for Autism Research, 3081 Quail Hollow, Sarasota, FL 34235 USA; Department of Psychiatry, AP-HP, Henri Mondor-Albert Chenevier Hospital, 40 rue de Mesly, 94000 Créteil, France; INSERM U955, Institut Mondor de Recherche Biomédicale, Psychiatric Genetics, 8 rue du Général Sarrail, 94000 Créteil, France; Faculty of Medicine, University Paris-Est Créteil, 8 rue du Général Sarrail, 94000 Créteil, France

## Abstract

**Background:**

Apparently balanced chromosomal rearrangements can be associated with an abnormal phenotype, including intellectual disability and autism spectrum disorder (ASD). Genome-wide microarrays reveal cryptic genomic imbalances, related or not to the breakpoints, in 25% to 50% of patients with an abnormal phenotype carrying a microscopically balanced chromosomal rearrangement. Here we performed microarray analysis of 18 patients with ASD carrying balanced chromosomal abnormalities to identify submicroscopic imbalances implicated in abnormal neurodevelopment.

**Methods:**

Eighteen patients with ASD carrying apparently balanced chromosomal abnormalities were screened using single nucleotide polymorphism (SNP) arrays. Nine rearrangements were *de novo*, seven inherited, and two of unknown inheritance. Genomic imbalances were confirmed by fluorescence in situ hybridization and quantitative PCR.

**Results:**

We detected clinically significant *de novo* copy number variants in four patients (22%), including three with *de novo* rearrangements and one with an inherited abnormality. The sizes ranged from 3.3 to 4.9 Mb; three were related to the breakpoint regions and one occurred elsewhere. We report a patient with a duplication of the Wolf-Hirschhorn syndrome critical region, contributing to the delineation of this rare genomic disorder. The patient has a chromosome 4p inverted duplication deletion, with a 0.5 Mb deletion of terminal 4p and a 4.2 Mb duplication of 4p16.2p16.3. The other cases included an apparently balanced *de novo* translocation t(5;18)(q12;p11.2) with a 4.2 Mb deletion at the 18p breakpoint, a subject with *de novo* pericentric inversion inv(11)(p14q23.2) in whom the array revealed a *de novo* 4.9 Mb deletion in 7q21.3q22.1, and a patient with a maternal inv(2)(q14.2q37.3) with a *de novo* 3.3 Mb terminal 2q deletion and a 4.2 Mb duplication at the proximal breakpoint. In addition, we identified a rare *de novo* deletion of unknown significance on a chromosome unrelated to the initial rearrangement, disrupting a single gene, *RFX3*.

**Conclusions:**

These findings underscore the utility of SNP arrays for investigating apparently balanced chromosomal abnormalities in subjects with ASD or related neurodevelopmental disorders in both clinical and research settings.

## Background

Balanced chromosome abnormalities, including translocations and inversions, are structural rearrangements of genetic material with no overall gain or loss detected with conventional karyotyping. Apparently balanced chromosomal rearrangements (ABCR) have an estimated frequency of 0.5% in newborns [1], including 14% *de novo*. About 6% of *de novo* ABCR detected at amniocentesis are associated with an abnormal phenotype, including intellectual disability (ID) and multiple congenital anomalies [2]. Because of the insufficient discrimination power of conventional cytogenetics, in which genomic imbalances smaller than 5 to 10 megabases (Mb) are usually not detected, the most common explanation for the clinical abnormalities is cryptic loss or gain of genomic material at or in the vicinity of the breakpoint. When there is no loss or gain of DNA sequences, the rearrangement can disrupt a dosage-sensitive gene, separate a gene from its *cis* regulatory elements, or generate a functional chimeric gene.

Studies using DNA microarray technologies have demonstrated submicroscopic anomalies related or not to the breakpoint in 46% (range 31% to 100%) of patients with an abnormal phenotype carrying a *de novo* ABCR [3-10]. Inherited ABCR in patients with an abnormal phenotype have been studied less often, but they can also be associated with cryptic imbalances at the breakpoint or elsewhere in the genome, with a combined frequency of 25% (6/24) in three studies [7-9]. Imbalances are more frequent in complex rearrangements involving more than two breakpoints and in patients with a complex phenotype [10]. In contrast, genomic imbalances are unlikely to be detected in phenotypically normal carriers of apparently balanced translocations [6]. Taken together, these findings indicate that a significant proportion of ABCR in phenotypically abnormal individuals are in fact associated with genomic imbalances and that these rearrangements should be systematically investigated by high-resolution microarrays independently of their *de novo* or inherited origin. The phenotypes of the patients in previous studies were very heterogeneous, including developmental delay, ID, multiple congenital anomalies, and autism spectrum disorder (ASD).

ASD is an etiologically heterogeneous neurodevelopmental disorder characterized by impairments in social communication and by restricted interests and stereotyped behaviors. Hundreds of rare variants, including chromosomal abnormalities, copy number variants (CNVs), and single nucleotide variants have been implicated in ASD [11,12]. However, for about 80% of cases, the underlying genetic determinants remain unknown. The frequency of structural chromosomal imbalances detected by conventional cytogenetics in autism varies between 2% and 6% [13,14], including karyotypically balanced chromosomal abnormalities. To date, only isolated cases with ABCR and ASD have been studied by array technology (for example [15,16]). Here we report a series of 18 patients with ASD carrying *de novo* or inherited ABCR studied by single nucleotide polymorphism (SNP) arrays to identify cryptic CNVs implicated in abnormal neurodevelopment.

## Methods

### Subjects

Patients with ASD and ABCR were ascertained through two sources: 1) 12 patients from the Paris Autism Research International Study (PARIS) family dataset [17], and 2) 6 patients referred by the network of French cytogeneticists. A summary of the clinical and cytogenetic data of the patients is shown in Table 1. Sixteen patients fulfilled DSM-IV criteria for autistic disorder, while two subjects (patients 9 and 13) had a previous diagnosis of ASD but could not be formally evaluated for ASD for this study. The Autism Diagnostic Interview-Revised (ADI-R) was performed in 14 subjects; in 1 individual, the Diagnostic Interview for Social and Communication Disorders, tenth revision (DISCO-10) was used instead. In addition, five subjects were assessed with the Autism Diagnostic Observation Schedule (ADOS). Fourteen patients had ID. Based on the presence of facial dysmorphism and/or malformations, six subjects were considered to have syndromic ASD (patients 3, 4, 5, 9, 10, and 15). Fragile X molecular testing and metabolic screening were normal in all individuals. All parents were phenotypically normal, including those carrying a rearrangement. The study was performed in accordance with the ethical standards of the responsible institutional and national committees on human experimentation, in compliance with the Helsinki Declaration. Informed consent was obtained from all families participating in the study. For the PARIS patients, the study was approved by the research ethics boards of the collaborating institutions (Comité de Protection des Personnes Ile-de-France VI, Paris, France, and Ethical Review Board in Gothenburg, Sweden).

**Table 1:** **Clinical and cytogenetic characteristics of patients**

**Patient**	**Initial karyotype**	**Sex**	**Age at last evaluation**	**ASD**	**Cognitive level**	**Language**	**Birth and early development**	**Body measures**	**Dysmorphic features**	**Other**	**Brain MRI**
1^a^	46,XY,der(4)t(4;acro p)(p16.3;acro p)dn	M	4 y	Autism (DISCO)	VIQ 67, PIQ 78, FSIQ 69 (WPPSI-R, 34 mo)	Functional language	Born at 42 wk, W +4.3 SD (gestational diabetes), neonatal hypoglycemia. Normal motor milestones	4 y: W and H +2-3 SD	No dysmorphic features, mild strabismus	Inguinal hernia	Not done
2^a^	46,XY,t(5;18)(q12;p11.2)dn	M	5 y	Autism (ADI-R)	DQ 52 (PEP-R, 40 mo)	1st words 36 mo, no sentences	Born at 38 wk, W –0.6 SD, H –1 SD, OFC mean. Walked at 17 mo	5 y: W –1 SD, H +1 SD, OFC –0.4 SD	No dysmorphic features, slender habitus, long fingers and toes, numerous secondary palmar creases	Frequent otitis, eczema	Small bilateral insular hypersignal suggesting myelinization delay (at 4 y)
3^b^	46,XY,inv(11)(p14q23.2)dn	M	4 y	Autism (ADI-R, ADOS)	DQ 13 mo (Brunet Lezine-R, 4 y)	Non-verbal	Intrauterine growth retardation, born at 37 wk, W –3.5 SD, H –3.5 SD, OFC –2.5 SD. Delayed motor development, unable to walk at 4 y	42 mo: W, H and OFC –3 SD	Mild dysmorphic features, high forehead, horizontal eyebrows, upslanting palpebral fissures, bulbous nose, smooth philtrum, thin upper lip, posteriorly rotated ears, single palmar crease, bilateral clinodactyly 5th finger	Aggressiveness, anxiety, stereotypies, head banging, bilateral cryptorchidism, axial hypotonia, hyperlaxity	Supratentorial ventricular enlargement, increased subarachnoid spaces (at 18 mo)
4^a^	46,XY,inv(2)(q14.2q37.3)mat (reported previously [15])	M	14 y	Autism (ADI-R)	VIQ 46, PIQ 50, FSIQ 46 (WISC-III, 14 y)	1st words 30 mo, 1st sentences 60 mo	Born at 39 wk, W mean, H –0.5 SD, OFC –1.1 SD. Sat at 9 mo, walked at 14 mo	12 y: W +0.5 SD, H –0.5 SD, OFC mean	Mild dysmorphic features, frontal bossing, flattened nasal bridge, deep-set eyes, downslanting palpebral fissures, thin upper lip	Hyperactivity, head banging, anxiety, asthma, insulin-dependent diabetes, growth hormone deficit	Normal (at 3 y)
5^a^	46,XX,inv(2)(p12q14.1)dn (benign cytogenetic variant)	F	13 y	Autism (ADI-R)	DQ 30 (PEP-R, 6 y)	Non-verbal	Born at 39 wk, W +0.5 SD, H mean, OFC +1.9 SD. Sat at normal age, walked at 22 mo	13 y: W +3.5 SD, H +2 SD, macrocephaly (+4.2 SD)	Mild dysmorphic features, narrow palpebral fissures, short philtrum, large hands and feet, one café-au-lait spot	Stereotypies, self-injurious behavior, sleep disturbance, one episode of febrile seizure at 31 mo, normal EEG	Chiari type I malformation, mild white matter hyperintensities (at 9 y)
6^a^	46,XX,inv(9)(p11q13)dn (benign cytogenetic variant)	F	7 y	Autism (ADI-R)	Intellectual disability	Isolated words	Born at term, W +0.5 SD, H +0.9 SD. Walked at 12 mo	7 y 2 mo: W +3.8 SD, H +4.1 SD, OFC +1.7 SD	No dysmorphic features	Precocious puberty at 7 y, bone age 8 y, normal hormone levels, pulmonar stenosis, moderate systolic murmur	Normal (at 7 y)
7^a^	46,XY,inv(5)(q13q34)dn	M	18 y	Autism (ADI-R)	VIQ 93, PIQ 80, FSIQ 86 (WISC-III, 18 y)	No delay, functional language	Born at 40 wk, mean W, H and OFC. Sat at 9 mo, walked at 20 mo	18 y: W –1.4 SD, H mean, OFC +1.6 SD	No dysmorphic features, mild clubbing	Refraction error	Myelinization delay (at 18 y)
8^b^	46,XY,t(9;19)(p12;q13.4)dn	M	7 y	Autism (ADI-R, ADOS)	VIQ 83, PIQ 80, FSIQ 79 (WPPSI, 5 y 5 mo)	1st words 24 mo, 1st sentences 36 mo, functional language	Born at term, W +1 SD, OFC +0.3 SD. Sat at 7 mo, walked at 11 mo	NA	No dysmorphic features	ADHD	Normal
9^b^	46,XX,t(X;5)(p11.2;q35.2)dn (reported previously [59])	F	8 y	ASD^c^	PIQ 58 (WISC-R at 8 y)	Non-verbal, uses sign language	Born at term, W +1 SD. Sat at 9 mo, walked at 21 mo	4 y: W and H mean, OFC +1 SD	Mild dysmorphic features, prominent forehead, saddle nose, midface hypoplasia, high arched palate, generalized alopecia with scattered thin hair, umbilical hernia, pectus excavatum	Hypomelanosis of Ito, pigmented lesions on the legs, achromic lesion on the back, hypotonia, hyperlaxity, flat feet, hypermetropia, febrile seizures, absence seizures, abnormal EEG. Skewed X inactivation of the normal chromosome (92%)	Normal (at 6 y)
10^a^	46,XY,t(20;21)(q11.2;q21)dn	M	12 y	Autism (ADI-R)	DQ 20 (PEP-R, 12 y)	Non-verbal	Born at 41 wk, W –1.7 SD, H –0.5 SD, OFC –1.1 SD. Sat at 9 mo, walked at 18 mo	12 y: W –1.8 SD, H mean, microcephaly (–3.6 SD)	Dysmorphic features, low forehead, thick eyebrows, long nose, short philtrum, right single transverse palmar crease	Strabismus, epilepsy (onset at 9 mo)	Not done
11^a^	46,XY,t(9;16)(q3.2;p1.2)mat	M	18 y	Autism (ADI-R)	VIQ 46, PIQ 46, FSIQ 40 (WISC-III, 14 y)	Language delay, 1st phrases 5 y, functional language	Born at term, W +1.3 SD, H +1 SD, OFC +2.1 SD. Sat at 8 mo, walked at 14 mo	18 y: W +3 SD, H mean, OFC +2.3 SD	No dysmorphic features, short neck, brachymetatarsia of 4th and 5th rays	Hyperactivity in childhood, strabismus, hypermetropia	Not done
12^a^	46,XY,t(3;8)(q13.2;p23)mat	M	5 y	Autism (DSM-IV, CARS)	VIQ 79, PIQ 74, FSIQ 77 (Stanford-Binet IV, 3 y 9 mo)	1st words 26 mo, 1st sentences 36 mo, few sentences at 5 y, dysarthric speech	Normal pregnancy, delivery and early development; walked at 13 mo	4 y: W, H and OFC within normal limits	No dysmorphic facial features, long ring finger, brachymesophalangia V, sandal gap	Severe ADHD, pica, self-injurious behavior, partial complex epilepsy (onset at 4 y, currently seizure free on medication), chronic ear infections, chronic diarrhea, food allergies	Not done
13^b^	46,XY,t(2;20)(q13;q13.33)mat	M	22 y	ASD^c^	Intellectual disability	Language delay, uses only a few words, answers with signs	Born at term, W –0.8 SD, H –1 SD, OFC –1.1 SD. Sat at 11 mo, walked at 2 y	22 y: W +1.4 SD, H mean, OFC –1 SD	No dysmorphic facial features, bilateral clinodactyly 5th finger, several café au lait spots	Hyperactivity, aggressiveness, inappropriate laughter, hand stereotypies, unilateral strabismus, seizures at 7 mo, no recurrence after stopping treatment	Normal (at 1 y)
14^a^	46,XX,inv(5)(p13.3q13.3)pat (benign cytogenetic variant)	F	8 y	Autism (ADI-R)	DQ 55 (PEP-R, 5 y)	1st words 18 mo, 1st sentences before 3 y; echolalic language	Dizygotic twin pregnancy, born at 27 wk, W 880 g. Walked at 18 mo	8 y: W mean, H and OFC +1 SD	No dysmorphic features, long face, flat feet, tuberous angioma scar on scapula	Insensitivity to pain	Non specific white matter hypersignal (at 5 y)
15^a^	46,X,inv(Y)(p11q11)pat (benign cytogenetic variant)	M	14 y	Autism (ADI-R)	DQ 15 (PEP-R, 14 y)	Non-verbal	Born at 38 wk, W –2.1 SD, H –1.5 SD, OFC –2 SD. Sat at 9 mo, walked at 13 mo	14 y: W +2 SD, H +1 SD, OFC mean	Synophris, abnormal dental implantation (delayed tooth loss)		Normal (at 14 y)
16^b^	46,XY,t(2;13)(p23;q14)pat	M	7 y	Autism (ADI-R, ADOS)	PIQ <1st %ile (Raven, 7 y 5 mo)	1st words 36 mo, 1st sentences 48 mo, functional language	Born at term, W –1.2 SD, H +0.5 SD, OFC +0.7 SD. Sat at 20 mo, walked at 36 mo	NA	No dysmorphic features	ADHD	Normal
17^a^	46,XY,t(4;9)(p13;p23) (not maternal, father not tested)	M	30 y	Autism (ADI-R, ADOS)	DQ <20 (PEP-R, 16 y)	Non-verbal	Born at term, W +0.7 SD, H +1 SD. Feeding difficulties, hypotonia, growth delay (–3 SD). Walked at 4 y	30 y: W –1 SD, H +1.1 SD, OFC –0.5 SD	No dysmorphic features except for enophtalmia, low-set eyebrows, large ears, narrow hands	Hyperactivity, severe sleep disturbance, insensitivity to pain, gastric and esophageal ulcers, abnormal EEG at 1 y (temporal lobe focus), no epilepsy	Not done (encephalography at 6 mo: global ventricular dilatation)
18^b^	46,XY,t(2;13)(q22;q31) (not maternal, father not tested)	M	4 y	Autism (ADI-R, ADOS)	IQ <1st %ile (Raven, 4 y 8 mo)	Non-verbal	Born post-term at 47 wk, W –0.8 SD, H –0.8 SD, OFC –0.6 SD. Sat at 20 mo, walked at 36 mo	4 y: W and H –0.5 SD, OFC –2 SD	No dysmorphic features, brachyplagiocephaly	ADHD, aggressiveness, anxiety	Normal

^a^Patients from the PARIS cohort; ^b^patients referred by the network of French cytogeneticists; ^c^ASD was not formally evaluated in these two patients. ADHD, attention deficit-hyperactivity disorder; ADI-R, Autism Diagnostic Interview-Revised; ADOS, Autism Diagnostic Observation Schedule; ASD, autism spectrum disorder; CARS, Childhood Autism Rating Scale; DISCO, Diagnostic Interview for Social and Communication Disorders; dn, *de novo*; DQ, developmental quotient; FSIQ, full scale IQ; H, height; IQ, intellectual quotient; mat, maternal; mo, months; MRI, magnetic resonance imaging; NA, not available; OFC, occipitofrontal circumference; pat, paternal; PEP-R, Psychoeducational Profile Revised; PIQ, performance IQ; SD, standard deviation; VIQ, verbal IQ; W, weight; WISC-III, Wechsler Intelligence Scale for Children, third edition; WISC-R, Wechsler Intelligence Scale for Children-Revised; wk, weeks; WPPSI-R, Wechsler Preschool and Primary Scale of Intelligence Revised; y, years.

### Conventional cytogenetic analysis

Karyotype (G-banded and/or R-banded, 450 band level) showing an ABCR was available for all patients and in most of the parents before inclusion. The sample included ten autosomal reciprocal translocations, one X-autosome translocation, three rare inversions, and four common pericentric inversions of chromosomes 2, 5, 9, and Y, usually considered as cytogenetic variants (Table 1). Nine chromosomal rearrangements were *de novo*, four were maternally inherited, three were paternal, and in two cases, the inheritance could not be defined because no blood samples were available from the fathers.

### Whole-genome SNP array

SNP array analysis was performed in affected individuals and their parents using the Human CNV370-Duo DNA Analysis BeadChip (Illumina, San Diego, CA, USA). In three cases, parental DNA was not available (patients 8, 9, and 18), and in one case, only the mother was available (patient 17). The array contains more than 370,000 markers, with a mean resolution of 30 kb. Genomic DNA was processed according to the Infinium II assay manual. SNP copy numbers (log R ratio) and B allele frequencies were assessed using the Bead Studio software version 3.2, with the CNV partition algorithm, V 1.3.2 or 2.4.4 (Illumina). All genomic coordinates are based on GRCh37/hg19. The clinical relevance of CNVs was interpreted according to the American College of Medical Genetics guidelines [18].

### FISH analyses

Fluorescence *in situ* hybridization (FISH) analysis was carried out using specific bacterial artificial chromosomes (BACs) to confirm CNVs larger than 1 Mb. BAC clones were hybridized on metaphase spreads of patients and their parents, in order to confirm the inheritance.

### qPCR

Real-time quantitative PCR (qPCR) was used to confirm and map CNVs smaller than 1 Mb. For patient 2, qPCR was used to confirm a larger CNV because metaphase spreads were not available. We used the Universal Probe Library (UPL) system (Roche, Indianapolis, IN, USA) and a LightCycler 480 real-time PCR system (Roche), as described previously [19].

## Results

In this study, 18 patients with ASD and an apparently balanced chromosome rearrangement were analyzed for submicroscopic imbalances using genome-wide SNP arrays (Table 1). The microarray analysis detected clinically significant *de novo* CNVs in 4/18 patients (22%), including 3/9 with *de novo* ABCR and 1/7 with an inherited abnormality (Table 2). Patients 1 and 4 had both a deletion and a duplication in the chromosome implicated in the rearrangement. Patient 2 had a deletion at one of the translocation breakpoints, whereas patient 3 carried a deletion on a chromosome unrelated to the known rearrangement. The size of the pathogenic CNVs ranged from 3.31 to 4.93 Mb. In addition, patient 5 carried a *de novo* deletion on a chromosome unrelated to the initial rearrangement and disrupting a single gene, *RFX3*. In the remaining 13 patients, no clinically relevant rearrangement was detected. None of the patients had long contiguous stretches of homozygosity involving multiple chromosomes.

**Table 2:** ***De novo*** **abnormalities detected by SNP array**

**Patient**	**Initial karyotype**	**Imbalance**	**Chromosomal region**	**Start** ^**a**^	**End** ^**a**^	**Size (bp)**	**Parental origin**
Patients with clinically significant abnormalities							
1^b^	46,XY,der(4)t(4;acro p)(p16.3;acro p)dn	Deletion	4p16.3	17,764	558,839	541,076	Paternal
		Duplication	4p16.2p16.3	577,581	4,812,859	4,235,279	Paternal
2	46,XY,t(5;18)(q12;p11.2)dn	Deletion	18p11.22p11.31	5,408,997	9,625,750	4,216,754	Maternal
3	46,XY,inv(11)(p14q23.2)dn	Deletion	7q21.3q22.1	97,043,362	101,977,945	4,934,584	Paternal
4^c^	46,XY,inv(2)(q14.2q37.3)mat	Deletion	2q37.3	239,735,269	243,044,147	3,308,879	Maternal
		Duplication	2q14.1q14.2	117,072,756	121,304,548	4,231,793	Maternal
Variant of unknown clinical significance							
5	46,XX,inv(2)(p12q14.1)dn	Deletion	9p24.2	3,334,203	3,513,286	179,084	Paternal

^a^Genomic position in hg19 coordinates; ^b^in patient 1, the 4p duplication is pathogenic; the deletion is considered benign; ^c^in patient 4, the 2q37 deletion is pathogenic; the clinical significance of the duplication is unknown.

### Patient 1

**Figure 1:**
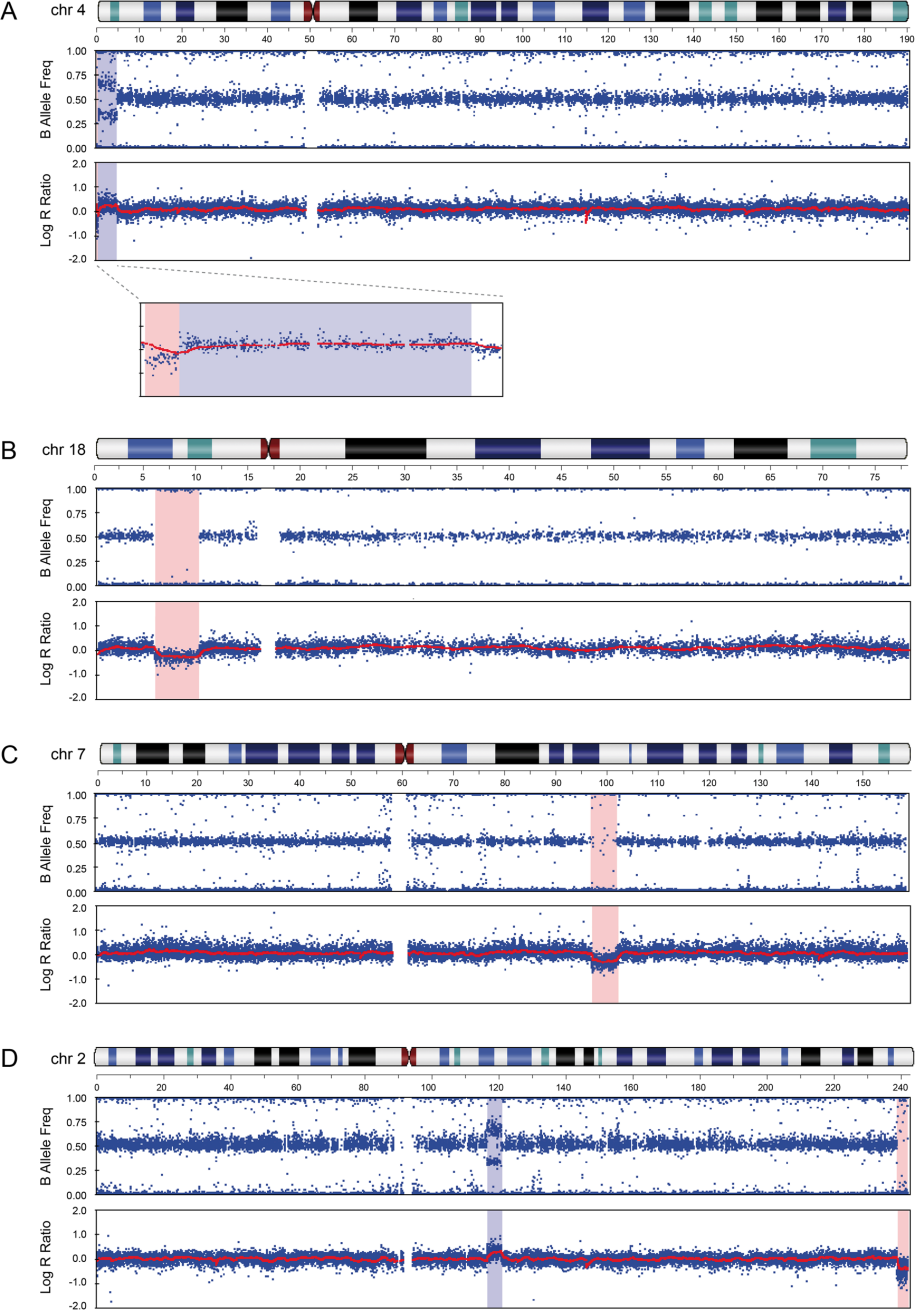
**SNP arrays of patients with clinically significant findings. (A)** Patient 1 (46,XY,der(4)t(4;acro p)dn) has a copy number loss of 541 kb in 4p16.3 and a gain of 4.29 Mb in 4p16.2p16.3. The distal deleted and duplicated 4p segments are shown in detail below. **(B)** Patient 2 (t(5;18)(q11.2;p11.2)dn) has deletion of 4.21 Mb in 18p11.31p11.22. **(C)** Patient 3 (46,XY,inv(11)(p14q23.2)dn) had no imbalance on chromosome 11 but the SNP array revealed a *de novo* deletion of 4.3 Mb in 7q21.3q22.1. **(D)** Patient 4 (46,XY,inv(2)(q14.2q37.3)mat) has a maternally inherited paracentric inversion of chromosome 2q, with a 2q14.1q14.2 duplication of 4.2 Mb at the proximal breakpoint, and a 2q37.3 deletion of 3.5 Mb extending to the telomere.

**Figure 2:**
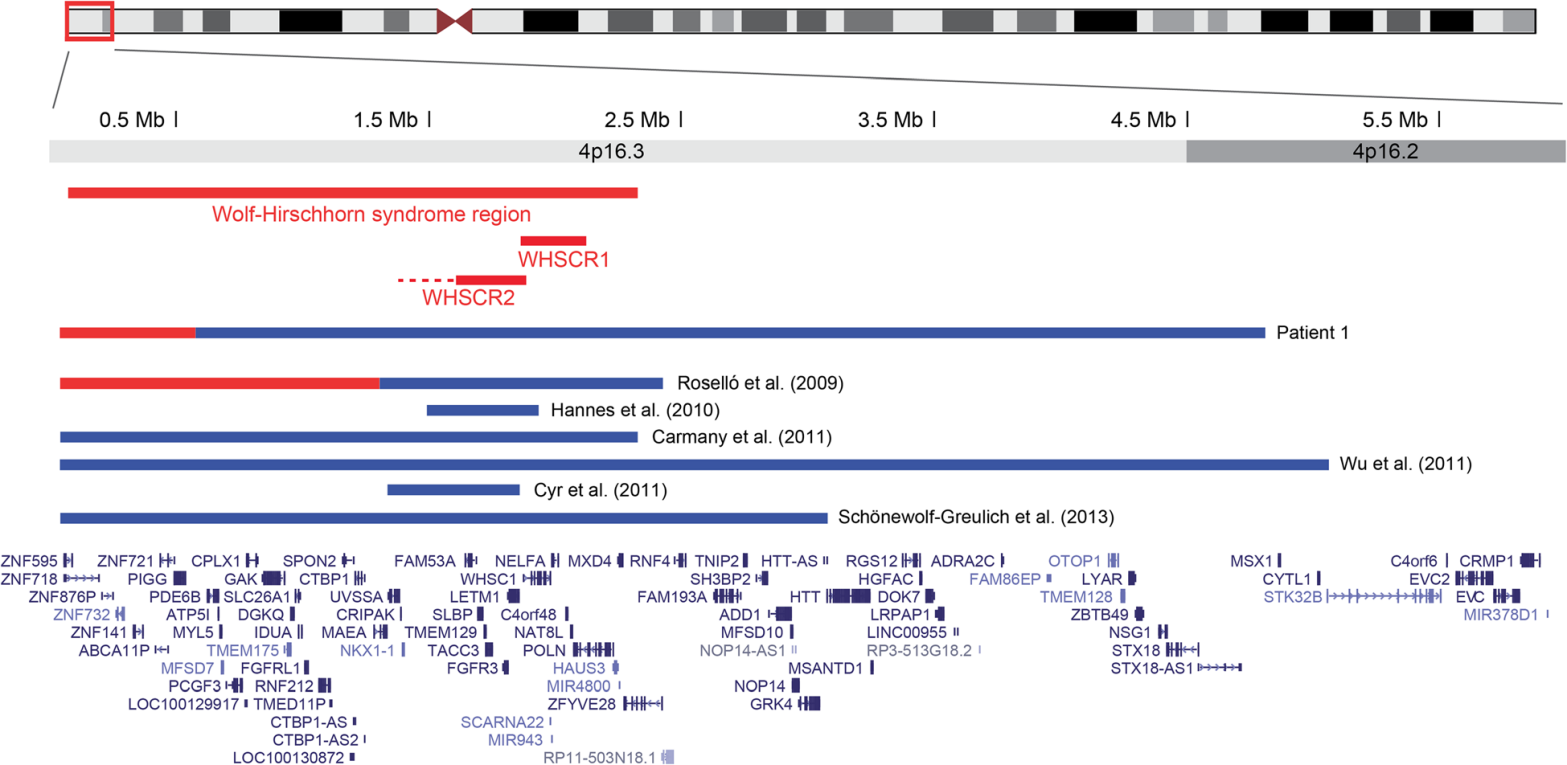
**Duplication of the Wolf-Hirschhorn region in patient 1.** Map of chromosome 4 (1-6,000,000, hg19) showing the rearrangement detected in patient 1 and previously reported overlapping rearrangements. The region commonly deleted in Wolf-Hirschhorn syndrome and the two proposed critical regions (WHSCR1 and WHSCR2) are represented. Horizontal blue lines represent duplications, and red lines indicate deletions. RefSeq genes are indicated at the bottom of the map.

This boy was referred for evaluation at the age of 2 years and 9 months because of developmental delay and ASD features. He is the second child of healthy non-consanguineous parents (maternal age 31 years, paternal age 38 years). He was born at 42 weeks after an uneventful pregnancy, weighing 5,280 g (+4.3 SD), probably due to latent diabetes in the mother. He was hypoglycemic during the first days of life, and an inguinal hernia was corrected at the age of two months. There was no delay in motor milestones or language development. He was diagnosed with autism after formal testing at an autism referral center. He had a borderline IQ of 69 (verbal IQ 67, performance IQ 78). There was no history of seizures, and his EEG was normal. His physical and neurological exams were normal except for mild strabismus; in particular, he had no dysmorphic features. At the age of 4 years, he was +2 SD to +3 SD in weight and height. Conventional karyotyping showed a *de novo* translocation between the telomeric region of the short arm of chromosome 4 and the short arm of an acrocentric chromosome. His karyotype was 46,XY,der(4)t(4;acro p)(p16.3;acro p)dn. Although this translocation is unbalanced, this patient was included in the present study because the excess of heterochromatic material from an acrocentric short arm cannot explain his abnormal phenotype. SNP array analysis revealed a 541 kb deletion in 4p16.3 (17,764-558,839, hg19) and a 4.24 Mb duplication in 4p16.2p16.3 (577,581-4,812,859) in the proband (Figure 1A), absent from the parents. Both rearrangements were confirmed by qPCR and FISH. The deletion was confirmed by FISH with the 4p subtelomeric probe RP11-2H3. Using probes RP11-296G16 (4p16.3) and RP11-265012 (4p16.2), the FISH findings were consistent with an inverted duplication (data not shown), indicating a chromosome 4p inverted duplication deletion. SNP genotyping indicated that both rearrangements occurred on the paternal chromosome. The deletion includes eight RefSeq genes; the duplication encompasses 70 genes and overlaps the Wolf-Hirschhorn critical region (Figure 2).

### Patient 2

The patient is a 5-year-old boy presenting with non-syndromic autism and mild ID. He is the second child of healthy non-consanguineous parents (maternal age 26 years, paternal age 36 years); antenatal and postnatal periods were uneventful. His language was delayed; he said his first words at 36 months and did not use sentences. He was diagnosed with autism following evaluation at the autism unit of a university hospital; he met criteria for autism according to the ADI-R. He suffered from recurrent otitis media in childhood and eczema. No dysmorphic features were noticed, except for long fingers and toes and numerous secondary palmar creases. Physical and neurological exams were normal. The original karyotyping showed a *de novo*, apparently balanced reciprocal translocation, 46,XY,t(5;18)(q12;p11.2)dn.ish t(5;18)(wcp5+;wcp5+,D18Z1+). The array revealed a 4.22 Mb deletion of chromosome 18p11.22p11.31 (5,408,997-9,625,750) (Figure 1B), originating on the maternally inherited chromosome. The deletion was confirmed by qPCR. Both parents had a normal array profile and normal gene dosage by qPCR.

### Patient 3

This boy is the first child of non-consanguineous healthy European parents (maternal age 32 years, paternal age 31 years) with unremarkable family history. Increased nuchal translucency was observed at 12 weeks of gestation. The amniocentesis karyotype was considered normal. At 22 weeks, an ultrasound revealed bilateral ectopic testes and dilated colon, and intrauterine growth retardation was noted at 32 and 36 weeks. He was delivered by cesarean section at 37 weeks because of fetal heart deceleration. He was transferred to the neonatal resuscitation unit, ventilated for 20 min, and placed in an incubator. Birth weight was 1,850 g (<5th centile), length 43 cm (<5th centile), and occipitofrontal circumference (OFC) 32.5 cm (<25th centile). Tests for congenital infections were negative. After 10 days, he was discharged with sequelae of congenital muscular torticollis. Feeding difficulties, axial hypotonia, and hyperlaxity were reported during the first months of life. At 10 months, his weight and length were below −2 SD, and his OFC decreased at −3 SD. His language development and motor milestones were delayed; he was able to stand up with support at 42 months and did not walk or talk at 4 years. When examined at the age of 42 months, his growth parameters remained at −3 SD. Dysmorphic features included high forehead, horizontal eyebrows, upslanting palpebral fissures, bulbous nose, smooth philtrum, thin upper lip, posteriorly rotated ears, single palmar crease, and bilateral clinodactyly of the fifth finger. Brain MRI showed ventricular enlargement and increased frontal subarachnoid spaces, suggesting global cerebral atrophy. The EEG and visceral and heart ultrasounds were normal. He was referred to a child psychiatry unit at the age of 4 years, where he received a diagnosis of autism based on DSM-IV criteria and confirmed by the ADI-R and ADOS. Evaluation with the Brunet-Lezine Revised Scale showed a developmental age of 13 months, indicating severe cognitive impairment. The Vineland Adaptive Behavior Scales showed developmental ages between 9 months (language) and 18 months (socialization). High-resolution karyotype revealed a *de novo* pericentric inversion of chromosome 11: 46,XY,inv(11)(p14q23.2)dn. The SNP array detected no imbalance on chromosome 11 but showed a *de novo* 7q21.3q22.1 deletion of 4.93 Mb (97,043,362-101,977,945) (Figure 1C), occurring on the paternal allele. The deletion was confirmed by FISH with BACs targeting 7q22.1 (RP11-44M6 and DBACA-20A02, Integragen, Evry, France) and excluded in the parents.

### Patient 4

The clinical and genetic characterization of this 14-year-old male was reported previously [15]. Briefly, he is the second child of a non-consanguineous healthy couple (maternal age 26 and paternal age 31 years) and presented with autism, ID, insulin-dependent diabetes, growth hormone deficiency, and mild dysmorphic features (Table 1). Standard karyotype showed an apparently balanced paracentric inversion of the long arm of chromosome 2 inherited from his healthy mother: 46,XY,inv(2)(q14.2q37.3)mat. Array analysis showed a 3.31 Mb terminal 2q deletion (239,735,269-243,044,147) at the distal breakpoint of the inversion and a 2q14.1q14.2 duplication spanning 4.23 Mb (117,072,756-121,304,548) at the proximal breakpoint (Figure 1D). Both imbalances occurred on the maternally derived chromosome and were confirmed by qPCR. FISH analysis revealed that the duplicated material was located at the telomeric end of chromosome 2, distal to the inverted region [15]. Both parents had normal SNP array profiles. The terminal deletion results in the 2q37 deletion syndrome, also known as brachydactyly mental retardation syndrome. Brachydactyly, reported in approximately 50% of affected individuals [20], was not observed clinically in patient 4.

### Patient 5

This female was referred at the age of 4 years and 8 months because of developmental delay, autistic behavior, and speech delay and diagnosed with autism after formal testing. She was born at term after an uneventful pregnancy, with weight and height in the normal range and OFC +1.9 SD. The parents were non-consanguineous; parental ages at the time of birth were 27 and 28 years for the mother and father, respectively. She walked at 22 months. At 3 years, she exhibited overgrowth, with height +3 SD and macrocephaly (+3 SD). Assessment of cognitive functioning at 6 years indicated severe ID. When re-evaluated at 11 years, she met criteria for autism according to the ADI-R, and the Vineland Adaptive Behavior Scales indicated developmental ages between 14 months (communication and socialization) and 37 months (motor skills). At the age of 13 years, all her growth parameters were increased (weight +3.5 SD, height +2 SD, OFC +4.2 SD), and she remained non-verbal. She had mild dysmorphic features, including narrow palpebral fissures, and short philtrum; her hands and feet were large, and a café-au-lait spot was present on a toe. A brain MRI performed at 9 years showed a Chiari malformation type 1 and mild hyperintensities of the white matter. The macrocephaly prompted screening for mutations and deletions in *PTEN*, with normal results [21]. The standard karyotype was 46,XX,inv(2)(p12q14.1)dn, which is considered a polymorphic cytogenetic variant. SNP array analysis revealed a *de novo* intragenic deletion of 179 kb in the *RFX3* gene at 9p24.2 (3,344,203-3,513,286), on the paternal allele. qPCR confirmed a deletion of exons 2 to 4 of *RFX3* in the patient and was normal in both parents.

## Discussion

We identified *de novo* genomic imbalances in 5/18 patients with ASD and ABCR, of which four were considered pathogenic (4/18, 22%) and one of unknown clinical significance. If we exclude the four patients carrying common pericentric inversions, which *a priori* are not expected to be causative of the phenotype, the yield increases to 33% (4/12). This is in accordance with a previous study showing that 24% of patients with abnormal phenotype and a *de novo* two-point chromosome rearrangement have a clinically significant cryptic genomic imbalance [10]. Three imbalances were related to the breakpoint regions, and two deletions occurred on unrelated chromosomes. In two patients, the SNP array analysis identified a terminal deletion and a duplication occurring on the same chromosome involved in the ABCR. Four out of five ABCR with a genomic imbalance occurred *de novo*: one rare pericentric inversion, one pericentric inversion of chromosome 2 considered as a chromosomal variant, and two reciprocal translocations*.* Two of the five patients with a *de novo* imbalance have a non-syndromic form of ASD. Our results are concordant with previous studies reporting that ABCR often hide more complex rearrangements regardless of the type of ABCR, particularly when they appear *de novo* [3,4,9,10], and when they are associated with an abnormal phenotype [3,5,6,8-10]*.* Of the five *de novo* imbalances, three originated on the paternally inherited chromosome, in agreement with previous studies showing that male gametogenesis is more susceptible to such rearrangements [4,6,9].

Four of our patients carry common inversions considered variants without phenotypic effects. In all cases, the inversion was indeed balanced, confirming that common pericentric inversions of chromosomes 2, 5, 9, and Y are not implicated in abnormal phenotype by imbalances at the breakpoints, even when they occur *de novo* [22-24]. In a patient with a pericentric inversion of chromosome 2, a *de novo* deletion occurred on an unrelated chromosome.

### Imbalances at the breakpoint region

#### Duplication of the Wolf-Hirschhorn region

Patient 1, carrying a translocation (4;acro p), has two imbalances, a 541 kb subtelomeric 4p16.3 deletion and a directly adjacent inverted duplication spanning 4.24 Mb. The 4p16.3 deletion overlaps two previously reported 4p terminal deletions between 200 and 400 kb in phenotypically normal individuals and can be considered benign [25,26]. The phenotype of our patient is likely related to the proximal duplication, which encompasses the Wolf-Hirschhorn critical region (WHSCR), with two proposed loci, WHSCR1 and WHSCR2 (Figure 2). Partial 4p trisomy, including the WHSCR, is associated with variable clinical manifestations depending on the size of the duplicated segment. Clinical features include growth retardation, ID, dysmorphic features, and heart and renal abnormalities. Most cases reported thus far were identified cytogenetically and are therefore very large [27-29]. Six smaller molecularly defined duplications overlapping the WHSCR have been reported in the literature (Figure 2). Roselló *et al*. [30] described a boy with a 1.1 Mb 4p16.3 duplication presenting with ID, dysmorphic features, language delay, absence seizures, hyperactivity, aggressive behavior, and non-specific anomalies on MRI. He also has a terminal 4p16.3 deletion of 1.3 Mb, interpreted as benign. A terminal 2.3 Mb duplication of 4p16.3 was reported by Carmany *et al*. [31] in a girl with mild phenotype, including delayed language development, borderline normal IQ, mild hypertelorism, and normal height with advanced bone age. The duplication resulted from a reciprocal translocation and was associated with a 314 kb deletion of 17q25.3, considered probably benign. A 5 Mb terminal 4p16.2 duplication, very similar to the one observed in our patient, was identified by Wu *et al*. [32] in two individuals from the same family with a t(4;15) translocation, a 2-month-old boy and his paternal uncle with mild ID and poor language. The two subjects shared some characteristics, including similar facial features, widely spaced nipples and small penis. Similarly, Schonewolf-Greulich *et al*. [33] described a 3 Mb terminal 4p16.3 duplication in a three-generation family with macrocephaly, tall stature, speech delay, mild ID, and minor dysmorphic features. Finally, two smaller interstitial duplications overlapping the WHSCR have been reported. Hannes *et al*. [34] detected an interstitial 4p16.3 duplication of 560 kb encompassing both WHSCR1 and WHSCR2 in a male toddler with developmental and speech delay, hypotonia, seizures, dysmorphic features, hand malformation, and glaucoma. The duplication occurred at the breakpoint of an inversion between 4p16.3 and 4q22; given the severity of his features, it is possible that the inversion contributes to his phenotype by disrupting a gene at the breakpoint. A 506 kb duplication in 4p16.3 involving only WHSCR2 was reported by Cyr *et al*. [35] in a 16-month-old boy with some characteristics of the trisomy 4p syndrome, including psychomotor delay, prominent glabella, low-set ears, and short neck. Compared to the patient reported by Hannes *et al*., the only common features observed in these individuals were developmental delay and seizures. Our patient presented with mild ID and autism, without malformations or seizures. Taken together, these individuals support the observation that duplications of the WHSCR result in variable, usually mild, clinical manifestations. The most common features include developmental delay, speech delay, and mild cognitive deficits; dysmorphic features, when present, are usually mild. ASD was not mentioned in any of the subjects with 4p16.3 duplications including the WHSCR or in patients with cytogenetically detectable duplications. In a large CNV study in ASD, Pinto *et al*. [36] identified a male with a 4p16.3p16.1 duplication spanning 9.3 Mb, but he also had a 6.8 Mb 8p23.3p23.1 terminal deletion resulting from a d*e novo* translocation. ASD has been reported in at least two patients with Wolf-Hirschhorn syndrome [37,38], suggesting that overexpressed genes within this region could be responsible for ID and ASD in our patient.

Inverted duplications with terminal deletions involving the short arm of chromosome 4 have been rarely reported in the literature [28,39-41]. The majority of rearrangements with terminal deletion associated with a duplication in the same chromosome arm correspond to inverted duplication deletions (inv dup del) [42]. Three mechanisms have been proposed to explain such rearrangements [42]. All involve an intermediate dicentric chromosome which breaks during meiotic division to produce a monocentric duplicated and deleted chromosome. The first mechanism occurs because of a parental paracentric inversion: during meiosis pairing, both homologues create an inversion loop; a crossing over in the loop leads to the formation of a dicentric chromosome and a reciprocal acentric chromosome. A subsequent breakage of the dicentric allows the formation of a monocentric chromosome with the inverted-duplicated-deleted chromosome. The second mechanism occurs through non-allelic homologous recombination between inverted segmental duplications in the same arm. For both mechanisms, the inverted duplicated region and the deleted region appear separated by a single copy region. The third mechanism, which is probably the most frequent, involves U-type exchange between the sister chromatids; in this case, the duplicated and deleted regions are adjacent and not separated by a single copy region [43,44]. In the patient reported here, there is no single copy region between the deletion and duplication, suggesting a U-type exchange mechanism. Moreover, as a telomere capture mechanism is frequently used for stabilizing the broken chromosome ends [44], we suggest that the translocation between the short arm of an acrocentric chromosome and the derivative chromosome 4 was generated to stabilize it.

#### 18p11.22p11.31 deletion

**Figure 3:**
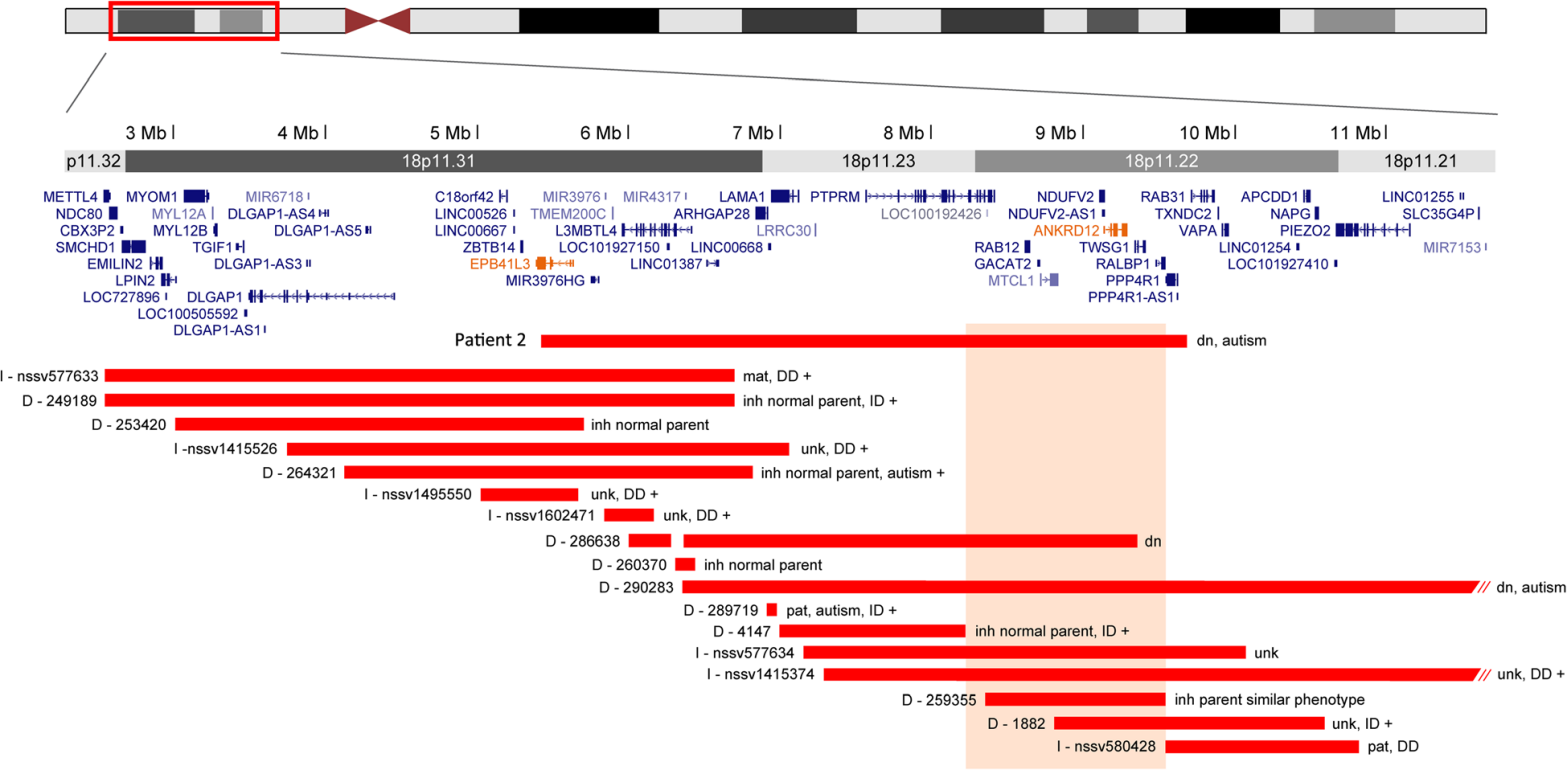
**18p11.31-p11.22 deletion in patient 2.** Map of chromosome 18 (2,500,000-11,700,000, hg19) showing the rearrangement detected in patient 2 and overlapping deletions from the DECIPHER (D) and ISCA (I) databases. The deletions are represented with horizontal red lines. RefSeq genes are indicated in blue. The phenotype is indicated when available. The + sign indicates additional morphological features. Two genes within the deleted region in patient 2 with a high haploinsufficiency score are indicated in orange. Most of the deletions in the distal region are inherited, usually from normal parents, whereas two other *de novo* deletions as well as one inherited from a parent with a similar phenotype overlap the proximal region, highlighted in peach. DD, developmental delay; dn, *de novo*; ID, intellectual disability; inh, inherited; mat, maternal; pat, paternal; unk, unknown inheritance.

In patient 2, with a *de novo* translocation (5;18), we identified an interstitial deletion of 4.22 Mb in the 18p11.22p11.31 region. The 18p deletion syndrome, associated with terminal deletions of variable size, is characterized by typical facial features, growth retardation, and ID [45]. Our patient does not have any dysmorphic features or growth retardation. Pure interstitial deletions in this region have not been reported in the literature so far. The DECIPHER and ISCA databases include several patients with partially overlapping interstitial deletions of variable sizes (Figure 3); although several are inherited from a normal parent, two are *de novo*, a 9 Mb deletion in a patient with autism (290283) and a 3 Mb deletion (286638, no phenotype information). The deletion in patient 2 overlaps 24 known genes; only 1 has a disease-associated OMIM entry, *NDUFV2*, involved in autosomal recessive mitochondrial complex I deficiency characterized by early-onset cardiomyopathy and encephalopathy. We speculate that haploinsufficiency of one or more genes included in the deletion could be implicated in the ASD phenotype. Two genes in the region have a high haploinsufficiency score, *EPB41L3* and *ANKRD12*, both highly expressed in brain*. EPB41L3* encodes band 4.1-like protein 3 (also known as protein 4.1B), which interacts with the synaptic cell adhesion molecule 1 (SynCAM1) to recruit NMDA receptors to synapses [46], and also acts as tumor suppressor. *ANKRD12* (ankyrin repeat domain 12) is a member of the ankyrin repeats-containing cofactor family, which can inhibit the transcriptional activity of nuclear receptors through the recruitment of histone deacetylases.

#### 2q37 terminal deletion

Patient 4 carries a maternal inv(2)(q14.2q37.3) with a terminal 2q37.3 deletion and a 2q14.1q14.2 duplication at the inversion breakpoints, which arose from a recombination event between the normal and inverted homologues 2 on maternal meiosis 1, followed by breakage of a dicentric chromosome [15]. The deletion involves the region implicated in the 2q37 deletion syndrome, frequently associated with ASD [38,47]. His phenotype is similar to that reported in patients with terminal 2q deletions and includes autism, ID, language delay, growth retardation, and mild facial dysmorphism. Haploinsufficiency of the *HDAC4* gene is responsible for brachymetaphalangy and ID [48], but the contribution of one or more distal genes cannot be excluded since individuals with distal deletions not including *HDAC4* have been reported with ID, ASD, and seizures. The 2q14.1q14.2 duplication includes 20 genes; no similar imbalances are reported in DECIPHER, ISCA, or the Database of Genomic Variants (DGV) so its clinical significance is unknown. Five smaller duplications with a common duplicated region of 440 kb (chr2:120,126,884-120,567,392) have been reported in DECIPHER; four are inherited from normal parents (one unknown inheritance), suggesting that they are probably benign.

### Imbalances on unrelated chromosomes

In patient 3, the *de novo* pericentric chromosome 11 inversion was not associated with a genomic imbalance but the SNP array analysis revealed a 7q21.3q22.1 *de novo* deletion of 4.9 Mb encompassing over 100 genes. In this interval, only *AP1S1* has been implicated in a neurological phenotype, a recessive disorder of copper metabolism. No similar deletions have been reported in the literature although several cases in DECIPHER and ISCA have smaller or larger deletions. Two inherited apparently balanced rearrangements mapping to this region have been reported in autism: a paracentric inversion (7q22.1q31.1) in two siblings with autism [49] and a translocation involving chromosome 7q22.1, just distal to *NPTX2*, in a subject with autism and ID [50].

Patient 5 has a pericentric inversion of chromosome 2, which is considered a benign cytogenetic variant. No CNV was observed in chromosome 2 but the SNP array identified a *de novo* 9p24.2 deletion interrupting the *RFX3* gene. A 440 kb deletion including *RFX3* was observed in one individual in a schizophrenia cohort [51]. Following-up on this finding, Sahoo *et al*. [52] analyzed 38,779 individuals referred for microarray testing and identified seven deletions encompassing the *RFX3* gene, with sizes ranging from 177 kb to 3.5 Mb. Two deletions were *de novo*, two paternal, and three of unknown origin. The clinical features included autism, ID, and behavioral problems. In addition, one DECIPHER patient (248290) has a partial deletion of *RFX3* but she also carries two large deletions in other chromosomes (no inheritance or phenotype information available). A similar deletion was reported in a patient from ISCA (nssv580652) with cataract and global developmental delay (unknown inheritance). DGV lists only one coding exon deletion (nsv892090), identified among 6533 Asians from the general population. Distal deletions of 9p of varying sizes are characterized by ID, trigonocephaly, dysmorphic facial features, and genital abnormalities; autism or autistic-like behavior are also common [53]. Two critical regions have been proposed for the 9p deletion syndrome, between 11 Mb and 16 Mb, and the first 2 Mb of 9pter [53], neither of which includes *RFX3* (located at 3.2 Mb). *RFX3* encodes a transcription factor required for ciliogenesis in mammals [54]. Many ciliopathy genes, such as those involved in Joubert syndrome, can present with neurodevelopmental phenotypes, including ID and ASD. *Rfx3*−/− mice show several hallmarks of ciliopathies, including left-right asymmetry defects, hydrocephalus, and corpus callosum agenesis; the phenotype of heterozygous mice was not reported [55,56]. Although the clinical significance of the *RFX3* deletion identified in our patient is unknown, the results on *Rfx3* mutant mice and the presence of rare *RFX3* deletions in patients with ID or abnormal behavior suggest that further studies are warranted on the contribution of *RFX3* loss of function to abnormal brain development.

## Conclusions

In this study, we used genome-wide microarray analysis to elucidate the molecular complexity of microscopically balanced chromosome rearrangements in a series of 18 patients with ASD. We detected *de novo* imbalances related to the initial ABCR or occurring elsewhere in the genome in 22%. This work provides additional evidence that ABCR are often more complex than they appear at first and can hide cryptic rearrangements involved in the abnormal phenotype regardless of the type and inheritance of the rearrangement. Thus, ABCR associated with abnormal phenotype have to be analyzed with genome-wide molecular cytogenetic techniques in order to exclude imbalances either within or outside the initial breakpoints. In the patients where a genomic imbalance was not detected by array analysis, higher resolution techniques based on next-generation sequencing [57,58] might be necessary to explore the rearrangement at the nucleotide level.

## Abbreviations

ABCR: apparently balanced chromosomal rearrangement
ADI-R: Autism Diagnostic Interview-Revised
ADOS: Autism Diagnostic Observation Schedule
ASD: autism spectrum disorder
BAC: bacterial artificial chromosome
CNV: copy number variant
DISCO-10: Diagnostic Interview for Social and Communication Disorders, tenth revision
FISH: fluorescence *in situ* hybridization
ID: intellectual disability
IQ: intellectual quotient
kb: kilobase
Mb: megabase
OFC: occipitofrontal circumference
qPCR: quantitative polymerase chain reaction
SNP: single nucleotide polymorphism
WHSCR: Wolf-Hirschhorn critical region
